# Insights into the Oxidative Stress Alleviation Potential of Enzymatically Prepared *Dendrobium officinale* Polysaccharides

**DOI:** 10.3390/molecules28073071

**Published:** 2023-03-30

**Authors:** Yingqi Tang, Xiong Zhang, Yudan Lin, Jiehan Sun, Shihao Chen, Weimin Wang, Jia Li

**Affiliations:** 1 College of Life Sciences, China Jiliang University, Hangzhou 310018, China; 2 Hangzhou Zaoxianyibu Food Technology Co., Ltd., Hangzhou 310018, China; 3 Hangzhou Jiuxian Biotechnology Co., Ltd., Hangzhou 311618, China; 4 Key Laboratory of Specialty Agri-Product Quality and Hazard Controlling Technology of Zhejiang Province, Hangzhou 310018, China

**Keywords:** *Dendrobium officinale* polysaccharide, enzyme-assisted extraction, oxidative stress resistance

## Abstract

(1) Background: The extraction parameters can dramatically alter the extraction rate and biological activity of polysaccharides. (2) Methods: Here, an enzyme-assisted extraction (EAE) was employed to extract *D. officinale* polysaccharides (DOPs), and its optimal extraction conditions were established by single-factor and Box–Behnken design (BBD) experiments. Further, on the basis of in vitro antioxidant capacity, the paraquat (PQ)-induced oxidative stress of *Caenorhabditis elegans* (*C. elegans*) was chosen as a research model to explore the antioxidant activity of DOPs. (3) Results: The results showed that the extraction yield of DOPs reached 48.66% ± 1.04% under the optimal condition. In vitro experiments had shown that DOPs have considerable ABTS^+^ radical scavenging capacity (EC50 = 7.27 mg/mL), hydroxyl radical scavenging capacity (EC50 = 1.61 mg/mL), and metal chelating power (EC50 = 8.31 mg/mL). Furthermore, in vivo experiments indicated that DOPs (0.25 mg/mL) significantly prolonged the lifespan, increased antioxidant enzyme activity, and upregulated the expression of daf-16 (>5.6-fold), skn-1 (>5.2-fold), and sir-2.1 (>2.3-fold) of *C. elegans*. (4) Conclusions: DOPs can be efficiently extracted by EAE and are effective in the reduction of oxidative stress levels in *C. elegans*.

## 1. Introduction

Oxidative stress is when the relative balance of reactive oxygen species (ROS) in the body becomes disturbed [1,2]. It can be the result of internal or external stimuli which have a damaging effect on biological macromolecules, such as proteins and nucleotides [3,4]. Scavenging excess ROS has been shown in several studies to reduce oxidative stress and delay the onset and progression of aging and related diseases [5].

Natural polysaccharides are considered to be promising in reducing oxidative stress injury [6]. Polysaccharides are known to have a wide range of biological activities, including anti-aging, immunomodulatory, hypolipidemic, hypoglycemic, and anti-tumor [5,7]. Notably, polysaccharides are well-absorbed by the body as well as being naturally biocompatible and non-toxic [8]. At present, the research on the antioxidant activity of natural polysaccharides is mainly focused on their ability to scavenge free radicals in vitro and their effects on the endogenous enzyme system against oxidative stress in vivo. The antioxidant activity of natural polysaccharides in vitro should be based on different antioxidant mechanisms and evaluated by different antioxidants, such as the radical scavenging rate, reducing power, and chelating capacity [9]. The study of antioxidation in vitro is simple and rapid, and the results can provide some reference for the study of antioxidation in vivo. On the other hand, natural polysaccharides alleviate oxidative stress in the body by regulating endogenous enzyme activities, mainly consisting of superoxide dismutase (SOD), catalase (CAT), and glutathione peroxidase (GSH-Px) [10].

*Caenorhabditis elegans* (*C. elegans*), *Drosophila*, and mammals are commonly used model organisms to study the mechanisms of oxidative stress mitigation. Among these, *C. elegans* has become an effective model for exploring potential mechanisms of oxidative stress and evaluating the antioxidant effects [11,12]. It has the advantages of being autotrophic, a short lifecycle, easy to cultivate, free from ethical restrictions [12,13], and 60~80% of genes shared with humans, including 12 signaling pathways [14,15]. In *C. elegans*, the oxidative stress-related insulin/IGF-1 signaling (IIS) pathway and the Nrf2 system are conserved, structurally and functionally [16,17,18,19]. Endogenous antioxidant enzymes enhance the oxidative stress barrier to ROS through the mediation of transcription factors (SKN-1 and DAF-16) [20,21]. In addition, enzyme expression at the mRNA level can be monitored by RNAi strains or qRT-PCR [22,23].

*Dendrobium officinale* Kimura et Migo (*D. officinale*) is an orchid plant with both medicinal and edible uses. *D. officinale* contains many bioactive components, including polysaccharides, polyphenols, flavonoids, and alkaloids, among which polysaccharides (DOPs) are the main one [24,25,26]. Zeng et al. [27] reported that DOPs reduced H_2_O_2_-induced apoptosis in human gastric mucosal epithelial cells by decreasing ROS levels and improving cell nuclear morphology. Huang et al. [28] found oxidative stress-alleviating activity of DOPs in cyclophosphamide-induced mice.

Historically, dendrobium polysaccharides have been prepared via hot water extraction, but it is an energy-intensive and time-consuming procedure. Therefore, ultrasonic, microwaves, acids, bases, and enzymes are used to assist extraction to improve extraction efficiency [26]. Enzyme-assisted extraction (EAE) is a gentler method than other extraction methods and is effective in breaking down cell walls and proteins to facilitate the release of polysaccharides. In past reports, cellulase-assisted extraction of DOPs has been demonstrated to enhance anti-inflammatory activity [29] and in vitro antioxidant activity [30]. Furthermore, it was found that complex enzymes (cellulose, papain, and pectinase)-assisted extraction enhanced the in vitro antioxidant activity of Astragalus polysaccharides [31]. Crude green tea polysaccharides exhibited effective hypoglycemic effects when extracted with the assistance of complex enzymes (cellulase, pectinase, and glucanase) [32]. The use of complex enzymes to extract DOPs and explore their role in alleviating oxidative stress damage is of great significance for the development of novel polysaccharide-based antioxidants.

In the current work, cellulase and papain were used to extract DOPs to improve the extraction efficiency of DOPs. A single-factor method and Box–Behnken design (BBD) were used to optimize the extraction conditions, such as extraction time, extraction temperature, enzyme concentration (E/S, enzyme weight: material weight), pH, and the ratio of liquid to material. Afterwards, the surface morphology of enzymatically extracted DOPs was characterized using scanning electronic microscopy (SEM). In addition, the in vitro antioxidant capacity of DOPs was evaluated by the ABTS^+^ radical rate, the hydroxyl radical scavenging rate, and the metal chelating power. Furthermore, their oxidative stress-relieving activity and the related mechanism were investigated using *C. elegans* under paraquat (PQ)-induced oxidative stress.

## 2. Results and Discussion

### 2.1. Single-Factor Experimental Analysis

**Figure 1:**
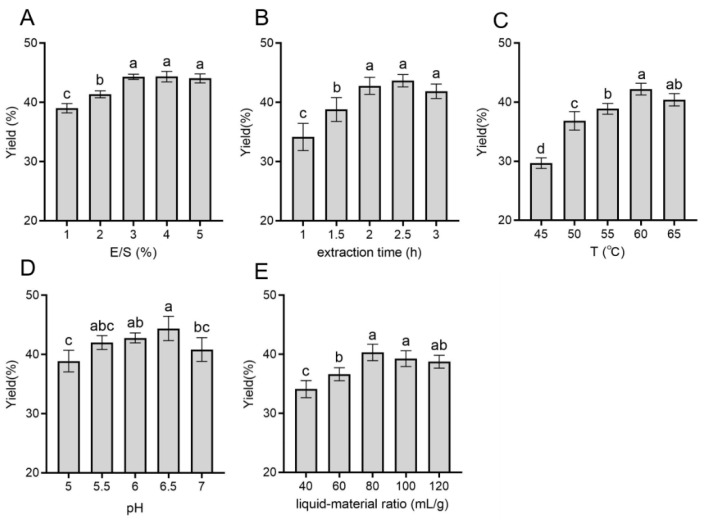
DOPs extraction yield at different (**A**) E/S, (**B**) extraction times, (**C**) extraction temperatures, (**D**) pH, and (**E**) liquid–material ratios. Extraction yield results were reported as means ± SD, and the bars with various letters (a–d) showed statistically significant differences (*p* < 0.05).

The effect of E/S on the extraction yield of polysaccharides is shown in Figure 1A. The extraction rate gradually increased from 38.02% ± 0.78% to 44.36% ± 0.40% with E/S increasing from 1% to 3%, and then leveled off, indicating that the polysaccharide dissolution of *D. officinale* reached the maximum degree when E/S was 3%. Figure 1B shows the effect of extraction time on the polysaccharide extraction yield. In the range of extraction time from 1 to 3 h, the extraction rate significantly increased, from 34.16% ± 2.30% to 43.66% ± 1.04%, after which the yield reduced to 41.94% ± 1.15%. Clearly, the extraction rate reached a maximum when the extraction time lasted for 2 h. In order to reduce the waste of resources and time, the extraction time was set to 2 h in subsequent experiments.

Figure 1C depicts that the extraction temperature plays a positive role in improving the extraction yield. The extraction rate was 29.69% ± 0.91% at 45 °C, and then reached 40.41% ± 1.05% at 60 °C. It was agreeable with some previous studies that polysaccharides were difficult to release and dissolve at low temperatures [33,34]. Although high temperatures (78~100 ℃) were favorable for collecting polysaccharides, too high temperatures might lead to degrading polysaccharides and further affect their chemical properties and biofunctions [29]. Thus, the optimum extraction temperature might be around 60 °C for EAE.

As shown in Figure 1D, the extraction rate obviously increased as the solvent pH climbed from 5 to 6. However, there was no marked difference at pH 5.5–6.5, even though the extraction rate reduced to 40.84% ± 1.99% at pH 7, the main reason for which was that acids and bases were usually used to enhance the release and solubilization of polysaccharides during extraction [35]. Additionally, the pH could influence the enzyme activity in this study, which was consistent with a reported conclusion [36]. Furthermore, according to Figure 1E, the extraction yield was significantly (*p* < 0.05) lower when the liquid–material ratio was less than 60 mL/g, and the optimal liquid–material ratio was probably around 80 mL/g.

The results of single-factor experiments demonstrated that the extraction temperature, pH, and the liquid–material ratio had greater effects on the yield of DOPs. This was consistent with the results of the factors influencing the cellulase-assisted extraction of polysaccharides from *Auricularia auricula* [37]. However, it was inconsistent with that of Pan et al. [29], who showed a more pronounced effect of pH and enzyme amount on the extraction yield, which might be due to different sample species and enzyme types [35]. Further optimization studies were required to determine the optimal extraction conditions for EAE extraction.

### 2.2. BBD Analysis

The yield of DOPs (Y) was utilized as the response values for the Box–Behnken design experiment. Based on single-factor analysis, a total of 17 optimizations were performed for 3 independent parameters: extraction temperature (A), liquid–material ratio (B), and solvent pH (C) (Table 1). The quadratic regression model equations for the results of the variables and the response values of the variables were obtained from the experimental results as follows:

(1) Y = 47.99 + 2.62A + 1.33B − (1.17C − 0.505AB − 2AC) − (−0.405BC − 2.16A2) − (4.77B2 − 3.43C2)

The analysis of variance (ANOVA) in Table 2 revealed that the F-value was 23.83 (*p* < 0.0002), suggesting that the model was highly significant, while the unfitting F-value of 1.48 (*p* = 0.3471) indicated a non-significant difference. In terms of the effect on extraction yield, all the primary terms were significant (*p* < 0.05) in the order A > B > C. Of the interaction terms, only AC was notable (*p* < 0.05). Besides, the secondary terms A_2_, B_2_, and C_2_ all had a significant effect (*p* < 0.01) on the extraction rate. Additionally, a high correlation between the variables and the response values was indicated by the coefficient of determination (R^2^ = 0.9684) and the adjusted coefficient of determination (Adj-R^2^ = 0.9277).

**Figure 2:**
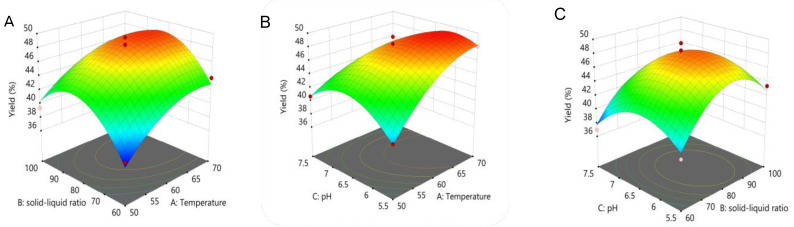
Three-dimensional (3D) response surface plots showing the effect of (**A**) AB, (**B**) AC, and (**C**) BC on the extraction yield of DOPs.

Figure 2 illustrates that there was a skew in the 3D response surface plot, whose degree of skewness is an indicator of how valid the experimental conditions are for the findings. According to the software analysis results, the suitable extraction temperature, liquid–material ratio, and pH were 67.794 °C, 82.31 mL/g, and 6.096, respectively, yielding a theoretical DOPs’ extraction rate of 49.33%. Subsequently, the experiment conditions were rechecked, and the actual extraction rate was 48.66% ± 1.04% (n = 3), which was in line with the anticipated yield and not statistically different (*p* > 0.05).

The DOPs’ yield can be influenced by factors such as origin, harvesting time, and extraction method. According to He et al. [30], the extraction method not only affects the yield of polysaccharides, but also the appearance, chemical properties, and antioxidant activity. It could be observed that the low viscosity of the extraction solution made it easier to separate from the residue using the optimized extraction condition. Polysaccharides obtained by enzymatic methods showed reduced MWs and improved bioactivities, i.e., probiotic, immunomodulatory, and antioxidant effects [38,39,40]. Therefore, further studies on the chemical composition and antioxidant activity could verify the feasibility of the EAE.

### 2.3. In Vitro Antioxidant Activity of Different Concentrations of DOPs

**Figure 3:**
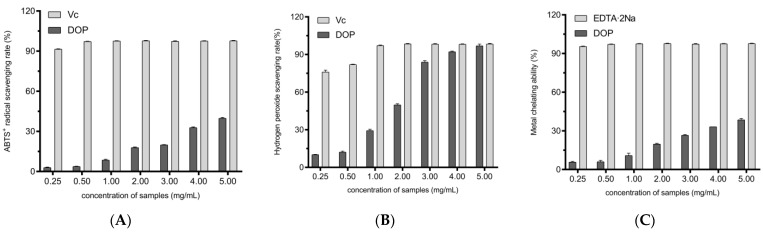
ABTS^+^ scavenging ability (**A**), hydroxyl radical scavenging ability (**B**), and metal chelating ability (**C**) for different concentrations of DOPs.

ABTS undergoes electron transfer to produce the stable green radical ABTS^+^. This is catalyzed by K_2_S_2_O_8_ to undergo electron transfer again to produce the stable radical ABTS^+^, which can be used to assess the antioxidant capacity [41]. Figure 3A shows the scavenging ability of different concentrations of DOPs on ABTS radicals. As the concentration of DOPs increased, the ABTS radical scavenging rate gradually increased to 39.82% ± 0.52% (EC50 = 7.27 mg/mL).

Hydroxyl radicals (·OH) are one of the types of ROS that are rapidly generated and can persist for a long period of time. They can directly damage proteins, causing significant damage to cells [42]. Thus, resistance to oxidative stress damage can be implied in in vitro experiments. As shown in Figure 3B, the scavenging ability of DOPs for hydroxyl radicals became stronger with the increasing concentration (EC50 = 1.61 mg/mL). At a concentration of 5 mg/mL of DOPs, the scavenging rate was as high as 96.95% ± 1.33%, which was not significantly different from Vc at the same concentration. Compared to the scavenging ability of ABTS^+^ radicals, DOPs showed a stronger scavenging ability of hydroxyl radicals.

Through the Fenton reaction, Fe^2+^ promotes the oxidation of lipids by splitting hydrogen and lipid peroxides to form reactive free radicals [43]. Ferrozine, a quantitative Fe^2+^ chelator, interferes with complex formation, resulting in a reduction in complex red color. Therefore, the reduction in color can be measured to assess the metal chelating activity of the coexisting chelator. As can be seen in Figure 3C, the metal chelating capacity of the DOPs decreased with increasing concentrations in relation to the positive control (EC50 = 8.31 mg/mL).

Free radicals are unstable groups of atoms. They constantly take electrons from other atoms and cause oxidation reactions. The ABTS radical scavenging ability of the DOPs was in agreement with that reported by Zhang et al. [44], while the hydroxyl radical scavenging ability was significantly higher. On the one hand, this may be due to differences in extraction methods, with enzyme-assisted extraction reported to enhance the antioxidant capacity of polysaccharides [30,31]. More importantly, this means that DOPs have the ability to scavenge ROS and to increase the activity of the SOD enzyme in vivo.

### 2.4. Effect of DOPs on the Longevity of Oxidative Stress Worms

**Figure 4:**
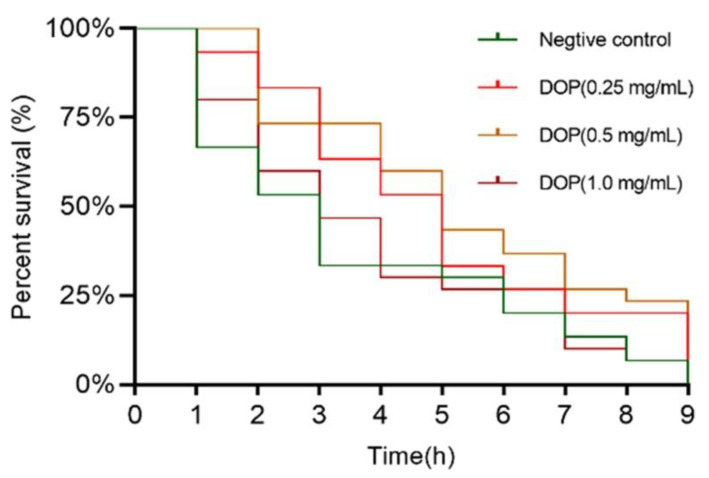
The effects of DOPs on the lifespan assay of *C. elegans* under PQ stress conditions.

When the stockpile of oxidation products is overwhelmed, a high amount of oxidative stress may trigger unintended harm [45]. As shown in Figure 4, worms in the negative control group showed significant mortality after one hour of stress. The survival rate of the low and medium DOPs (0.25, 0.5 mg/mL) was still as high as 50% after four hours of stress, while the survival rate of the negative control and high-concentration groups was only about 25%. It can be seen that high concentrations of DOPs lost their mitigating effect on PQ-induced oxidative stress. As shown in Table 3, the maximum survival time of nematodes in the negative group was 9 h, and the mean survival time was only 3.57 ± 0.5 h. All concentrations of DOPs significantly increased the mean survival time of worms under PQ-induced oxidative stress, extending the maximum survival time to 10 h. DOP (0.5 mg/mL) significantly prolonged the mean survival time of nematodes to 5.37 ± 0.49 h, with a median survival time of 3.88 ± 0.47 h. However, in the high DOPs (1.0 mg/mL) group, worms only had a mean survival time of 3.88 ± 0.47 h and a median survival time of 3 ± 0.61 h (*p* > 0.05). These results demonstrate that DOPs have a protective effect against PQ-induced oxidative stress.

### 2.5. Effect of DOPs on Biochemical Indicators

**Figure 5:**
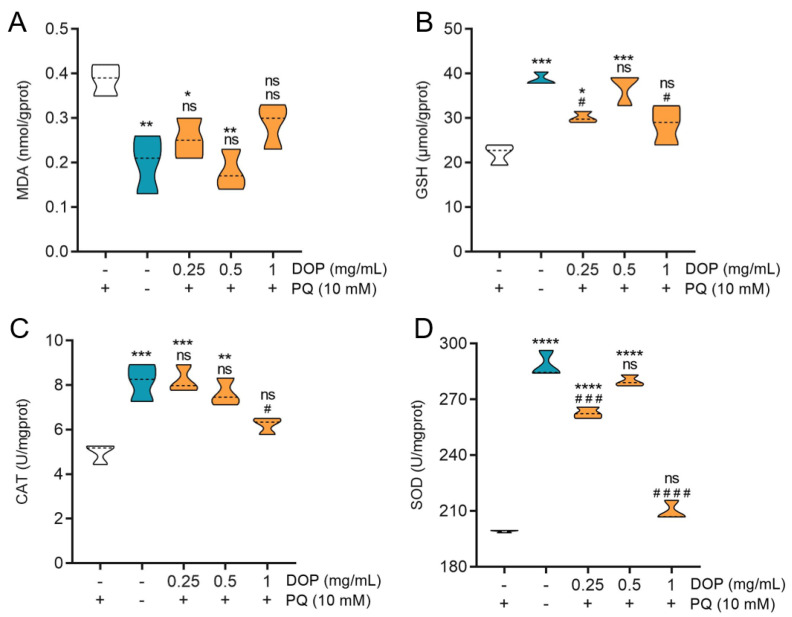
(**A**) MDA levels, antioxidant enzyme activities, (**B**) GSH levels, (**C**) CAT, and (**D**) SOD in the worms under PQ stress. Data are presented as mean ± SD (* *p* < 0.05, ** *p* < 0.01, *** *p* < 0.001, **** *p* < 0.0001, as compared with negative control worms, # *p* < 0.05, ### *p* < 0.001, #### *p* < 0.0001, as compared with normal feeding group worms; ns: no significance, hereafter).

Compared to the control group, PQ stress caused acute oxidative damage in the worms. This was manifested at the level of biochemical indicators as a significant increase in malondialdehyde (MDA) levels, a decrease in glutathione (GSH) levels, and a significant decrease in SOD and CAT enzyme activities (Figure 5). These results indicated the successful establishment of the nematode oxidative stress model. The low, medium, and high concentrations of DOPs’ pretreatment could all alleviate the oxidative stress, with MDA levels decreasing by 34.48%, 53.45%, and 25.86%, respectively. The CAT level in the worms treated at low concentrations was not significantly different from the normal feeding group. The SOD activity of the worms reached 279.90 ± 2.47 U/mgprot and the GSH content was 38.02 ± 2.97 μmol/gprot under the medium-concentration DOPs’ pretreatment.

The MDA level represents the extent of free radical cell damage, while the level of SOD and CAT activity shows the body’s ability to scavenge oxygen radicals [46,47]. The superoxide anion can be converted into hydrogen peroxide by SOD, while the hydrogen peroxide can be converted into oxygen by CAT, forming an antioxidant chain. Thus, they have been frequently employed to determine the antioxidant activity of polysaccharides in vivo in recent years [37,48]. GSH is the major biological free radical scavenger and antioxidant in the body, reflecting the redox status of cells [49]. The targets of oxidative stress triggered by PQ are complex and involve the disintegration of antioxidant systems. As a part of the adaptive response, the weakening of enzymatic (SOD, CAT activity) and non-enzymatic (GSH levels) defenses together suggests that PQ causes a harmful imbalance between internal reduction and oxidation. These results suggest that DOPs-feeding could relieve oxidative stress by improving the disorganization of antioxidant defense barriers.

### 2.6. Effects of DOPs on the Expression of Stress-Related Genes

**Figure 6:**
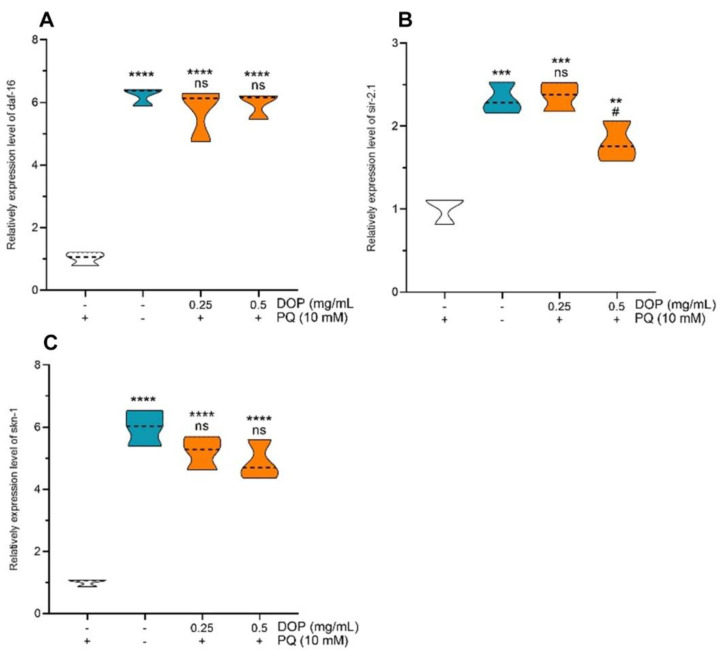
Effects of DOPs on the expression levels of (**A**) daf-16, (**B**) sir-2.1, and (**C**) skn-1 of C. *elegans.* Data are presented as mean ± SD (n = 3, ** *p* < 0.01, *** *p* < 0.001, **** *p* < 0.0001; # *p* < 0.05; ns: no significance) of three independent experiments.

As evident from Figure 6, the expression levels of daf-16, skn-1, and sir-2.1 were relatively lower in the negative control worms cultured under the oxidative stress conditions generated by PQ compared to the normal feeding group. The expression of daf-16 and skn-1 in *C. elegans* receiving 0.25 and 0.5 mg/mL of DOPs was significantly (*p* < 0.001) upregulated compared with the negative control group and not significantly different compared to the normal feeding group. When treated with DOPs at 0.25 mg/mL, the expression levels of daf-16, skn-1, and sir-2.1 were 5.73-, 2.36-, and 5.20-fold more than those of the negative control group. The DOPs (0.5 mg/mL) group showed an obvious 1.80-fold increase in sir-2.1 expression compared to the negative control group (*p* < 0.01).

The IIS pathway is the first documented aging regulatory system, and it is a crucial regulator of life activity, a process that is largely conserved between *C. elegans* and humans [50,51]. In *C. elegans*, daf-16 and skn-1 are two classical transcription factors involved in the IIS pathway [52], with the daf-16 belonging to the FoxO family of Forkhead transcription factors and the skn-1 transcription factor being functionally and physically similar to human NRF-2 [53,54,55]. Both of them participate in the stress response and minimize the oxidative damage in *C. elegans*. In addition, the sir-2.1 gene extends its lifespan in a daf-16/FoxO-dependent manner [56]. According to our findings, DOPs’ pretreatment upregulated the expression levels of major transcription factors on the IIS pathway in *C. elegans* (wildtype). Furthermore, these genes were involved in controlling stress responses, shielding proteins from toxic stress, thereby increasing the antioxidant capacity. More research is needed to confirm how DOPs impact the expression of oxidative stress-related proteins and reveal the pathways that defend against oxidative stress.

## 3. Materials and Methods

### 3.1. Materials and Chemicals

*D. officinale* was purchased from Hangzhou Jiuxian Biological Technology Co., Ltd. in Qiping Village, Lianhua Town, Jiande City, Hangzhou, Zhejiang, China. The fresh Dendrobium stems were cut into small segments and dried at 60 °C until constant weight. Then, they were crushed to 100 mesh. The *C. elegans* strains (N2) and *Escherichia coli* (OP50) were purchased from SunyBiotech Co., Ltd. in Fujian, China. Cellulase (10,000 U/g) and papain (200 U/mg) were purchased from Shanghai Macklin Biochemical Co., Ltd. (Building 1, 68 Huatuo Road, Zhangjiang High Tech Park, Pudong, Shanghai, China). All other reagents were of analytical grade and purchased commercially. Deionized water was utilized throughout the experiment.

### 3.2. Single-Factor Experiment and Response Surface Analysis Optimization

EAE was carried out using a complex enzyme (cellulase mixed with papain in equal proportions) with E/S of 4% at 50 °C for 2 h. Additionally, the extraction solvent was distilled water and the liquid–material ratio was 100 mL/g, with pH of 6. After the extraction, the enzyme was inactivated in a boiling water bath for 10 min and immediately filtered to obtain the *D. officinale* extract. The measurement of total sugar content was carried out according to a previous report [37], with D-glucose as the standard. The impacts of the liquid–material ratio (40, 60, 80, 100, 120 mL/g), extraction time (1, 1.5, 2, 2.5, 3 h), E/S (1, 2, 3, 4, 5%), pH (5, 5.5, 6, 6.5, 7), and extraction temperature (45, 50, 55, 60, 65 °C) were analyzed with *D. officinale* polysaccharide extraction yield as the dependent variable. 

To optimize the EAE process, the Box–Behnken design was carried out based on the results of single-factor experiments. The three individual factors affecting the polysaccharide extraction yield of *D. officinale* were coded as three levels (−1, 0, 1), resulting in a 17-run experimental design (Design-expert 12 software). 

DOPs were collected and low-temperature insoluble substances were removed according to a previous report [57]. The Sevag reagent was used to deproteinize the materials, and then the samples were sufficiently shaken to precipitate the protein layer, followed by retaining the supernatant after standing. This procedure was repeated until the solution was protein-free after centrifugation. The supernatant was lyophilized to obtain refined DOPs.

### 3.3. Antioxidant Activity Assays of DOPs In Vitro

#### 3.3.1. ABTS^+^ Scavenging Activity

The ABTS^+^ scavenging activity method was based on the reported method [58]. Here, 1.0 mL of the sample (5.0, 4.0, 3.0, 2.0, 1.0, 0.5, and 0.25 mg/mL) was added to 4 mL of diluted ABTS reserve solution, and absorbance was measured at 734 nm after 6 min. Vitamin C (Vc) was used as a positive control. The following equation was used to calculate the ability to scavenge the ABTS^+^ radical:

(2) $$\mathrm{Scavenging}\;\mathrm{rate}\;\left(\%\right)=\left(A_{blank}-A_{sample}\right)/A_{blank}\times 100$$

where $A_{blank}$ is the absorbance of anhydrous ethanol as the test sample, and $A_{sample}$ is the absorbance of DOPs or Vc.

#### 3.3.2. H_2_O_2_ Scavenging Activity

The disclosed approach was used to assess the H_2_O_2_ scavenging activity of DOP [59]. In short, DOPs concentration gradient solution (1 mL, 0.25 to 5 mg/mL) was combined with PBS (2.4 mL, pH of 7.4) and H_2_O_2_ solution (1 mL, 2 mM). After 30 min of incubation at 37 °C, the absorbance of the combination was measured at 230 nm. The H_2_O_2_ scavenging activity was determined using the following formula:

(3) $$\mathrm{Scavenging}\;\mathrm{rate}\;\left(\%\right)=\left(A_{blank}-A_{sample}+A_{background}\right)/A_{blank}\times 100$$

where $A_{blank}$ is the absorbance with water as the test sample, $A_{sample}$ is the absorbance of DOPs or Vc, and $A_{background}$ is the absorbance of the tested sample free of H_2_O_2_ solution.

#### 3.3.3. Assay of Ferrous Metal Ions’ Chelating Activity

The previously reported approach was used to assess the chelating activity of ferrous metal ions [60]. Here, 1 mL solutions with different DOPs concentrations (0.25 to 5 mg/mL) were mixed with FeCl_2_ solution (0.1 mL, 2.0 mM), ferrozine (0.2 mL, 5.0 mM), and water (3.7 mL). The mixture was set aside for ten minutes. The absorbance of the mixture was then measured at 562 nm. The following formula was used to calculate the ability of inhibiting ferrozine-Fe^2+^ complex formation:

(4) $$\mathrm{Metal}\;\mathrm{chelating}\;\mathrm{ability}\;\left(\%\right)=\left(A_{blank}-A_{sample}+A_{background}\right)/A_{blank}\times 100\;$$

where $A_{blank}$ is the absorbance with water as the test sample, $A_{sample}$ is the absorbance of the test samples or the positive control DETA·2Na, and $A_{background}$ is the absorbance of the tested sample in the absence of FeCl_2_ solution.

### 3.4. The Lifespan Assay of C. elegans under Oxidative Stress Conditions

According to the reported approach [61], before pouring the plates, filter-depleted 5-fluorouracil (50 mM) was added to the NGM medium to inhibit the production of zygotic worms. Worms at L4 phase were randomly transferred to plates containing DOPs (0.25, 0.5, 1.0 mg/mL) or M9 buffer and incubated continuously at 20 °C for 48 h. Groups of worms to be tested were transferred to 96-well plates containing paraquat (PQ, 200 μL, 70 mM) using a picker (~30 worms per group) and incubated at 20 °C. Death was determined by stiffness and unresponsiveness to touch, and the number of dead worms was counted every hour.

### 3.5. Oxidative Stress and Antioxidant Biomarker Biochemical Measurement

Referring to the method of Li et al. [62], a negative control group, a blank control group, and sample groups (low, medium, and high concentrations of DOPs: 0.25, 0.5, and 1.0 mg/mL, respectively) were set up in this experiment. Worms (4 plates per group) were collected, and then exposed to PQ (10 mM) and incubated at 20 °C for 2.5 h. Biochemical parameters were determined, including MDA concentration, GSH content, and the activity of the endogenous antioxidant enzymes SOD and CAT. Refer to commercially available kits (SOD (A001-3-2), CAT (A007-1-1), GSH (A006-2-1), total protein (A045-3-2), and MDA (A003-1-2) assay kits from Nanjing Jiancheng Institute of Biological Engineering (Nanjing, China)) for instructions.

### 3.6. Measurement of Oxidative Stress-Related Genes’ Expression

L4-phase worms were pretreated with DOPs solutions (0.25, 0.5 mg/mL) or M9 buffer for 48 h at 20 °C. Total RNA extraction and cDNA synthesis were performed according to previously reported methods [63]. The qRT-PCR detection was performed using SYBR^®^ Premix Ex Taq^TM^ II (TaKaRa, Japan), with β-actin as the internal reference gene, and the primer sequences are shown in Table 4. Primers were constructed based on NCBI sequences and designed at Hangzhou Youkang Biotechnology Co., Ltd., Hangzhou, China. The relative change in gene expression was calculated with the 2^−ΔΔCt^ methodology as follows:

(5) $$\Delta \Delta Ct={\left(Ct_{target}-Ct_{actin}\right)}_{sample}-{\left(Ct_{target}-Ct_{actin}\right)}_{control}$$

### 3.7. Statistical Analysis

All studies were carried out in triplicate, and the results were reported as mean ± standard deviation (mean ± SD). GraphPad Prism 8.0 and IBM SPSS 20 software were used to conduct one-way ANOVA tests for inter-group comparison. *p* < 0.05 was considered statistically significant.

## 4. Conclusions

In this study, DOPs were extracted using a complex of cellulase and papain enzymes. Through the response surface optimization, an optimized process for the preparation of DOPs was obtained: the E/S of 3%, the extraction time of 2 h, the extraction temperature of 68 °C, the liquid–material ratio of 82 mL/g, and pH 6, which corresponded to an extraction yield of 48.66% ± 1.04%. The ability to scavenge ABTS^+^ radicals and hydroxyl radicals as well as the metal chelating power suggest that DOPs have the potential to scavenge excess ROS and to alleviate lipid peroxidation. The oxidative stress damage induced by PQ in *C. elegans* was alleviated by the intervention of DOPs (0.5 mg/mL). The mean lifespan of *C. elegans* was prolonged by 50.42%, MDA content was decreased by 53.45%, and GSH content was increased by 167.93%, indicating that DOPs have an inhibitory effect on oxidative stress damage. The increase in SOD and CAT enzyme activities demonstrated that DOPs have a regulatory effect on endogenous antioxidant enzymes. In addition, DOPs upregulated the mRNA levels of the transcription factors daf-16 (5.8-fold), skn-1 (4.9-fold), and sir-2.1 (1.8-fold). In conclusion, cellulase and papain-assisted extraction can effectively increase the yield of *D. officinale* polysaccharides and the potential for functional food production.

## Figures and Tables

**Table 1:** The response values of DOPs’ extraction.

	A. Extraction Temperature (°C)	B. Liquid–Material Ratio (mL/g)	C. pH	Extraction Yield (Y) (%)
1	1 (70)	0 (80)	1 (7.5)	41.67
2	1	0	−1 (5.5)	48.11
3	1	1 (100)	0 (6.5)	43.88
4	0 (60)	−1 (60)	1	36.98
5	−1 (50)	−1	0	37.24
6	0	0	0	48.43
7	0	−1	−1	38.41
8	0	0	0	46.74
9	−1	0	−1	39.13
10	0	1	−1	43.41
11	0	0	0	47.92
12	1	−1	0	43.75
13	−1	1	0	39.39
14	0	0	0	49.48
15	0	1	1	40.36
16	−1	0	1	40.69
17	0	0	0	47.4

**Table 2:** ANOVA for response surface quadratic model.

Source	Sum of Squares	df	Mean Square	F-Value	*p*-Value
Model	279.85	9	31.09	23.83	0.0002
A	54.92	1	54.92	42.08	0.0003
B	14.2	1	14.2	10.88	0.0131
C	10.95	1	10.95	8.39	0.0231
AB	1.02	1	1.02	0.7816	0.4060
AC	16	1	16	12.26	0.0100
BC	0.6561	1	0.6561	0.5027	0.5012
A_2_	19.64	1	19.64	15.05	0.0061
B_2_	95.78	1	95.78	73.39	<0.0001
C_2_	49.67	1	49.67	38.06	0.0005
Residual	9.14	7	1.31		
Lack of Fit	4.81	3	1.6	1.48	0.3471
Pure Error	4.33	4	1.08		

R^2^ = 0.9684, Adj-R^2^ = 0.9277, C.V.% = 2.65.

**Table 3:** The effects of DOPs on the lifespan of *C. elegans* under oxidative stress conditions.

	Mean Survival Time (h)	Median Survival Time (h)	Max (h)	n
Negative control	3.57 ± 0.50	3 ± 0.52	9	42
DOPs (0.25 mg/mL)	4.93 ± 0.47 **	5 ± 0.57 **	10	38
DOPs (0.5 mg/mL)	5.37 ± 0.49 **	5 ± 0.54 **	10	35
DOPs (1.0 mg/mL)	3.88 ± 0.47 *	3 ± 0.61 ^ns^	10	31

Mean survival time and median survival time are presented as means ± SD (n = 29~42). * *p* < 0.05, ** *p* < 0.01, ns: no significance.

**Table 4:** Real-time quantitative PCR primers for antioxidant genes of *C. elegans*.

Gene	Forward 5′	Reverse 5′
β-actin	CCAGGAATTGCTGATCGTATGCAGAA	TGGAGAGGGAAGCGAGGATAGA
daf-16	GGAAGAACTCGATCCGTCACA	GATTCCTTCCTGGCTTTGCA
skn-1	TTCGCCTTCTCTCGAGGATATC	AACGTCTGCAAATCACATTCGT
sir-2.1	AAATCTTCCCAGGACAGTTCGTA	ATGGGCAACACGCATAGCA

## Data Availability

The authors confirm that the data supporting the findings of this study are available within the article.

## Sample Availability

Samples of polysaccharides are available from the authors.
